# Nanodiamond-enhanced MRI via *in situ* hyperpolarization

**DOI:** 10.1038/ncomms15118

**Published:** 2017-04-26

**Authors:** David E. J. Waddington, Mathieu Sarracanie, Huiliang Zhang, Najat Salameh, David R. Glenn, Ewa Rej, Torsten Gaebel, Thomas Boele, Ronald L. Walsworth, David J. Reilly, Matthew S. Rosen

**Affiliations:** 1 A.A. Martinos Center for Biomedical Imaging, Suite 2301, 149 13th Street, Charlestown, Massachusetts 02129, USA; 2 ARC Centre of Excellence for Engineered Quantum Systems, School of Physics, University of Sydney, Sydney, New South Wales 2006, Australia; 3 Department of Physics, Harvard University, 17 Oxford Street, Cambridge, Massachusetts 02138, USA; 4 Harvard Medical School, 25 Shattuck Street, Boston, Massachusetts 02115, USA; 5 Harvard-Smithsonian Center for Astrophysics, 60 Garden Street, Cambridge, Massachusetts 02138, USA

## Abstract

Nanodiamonds are of interest as nontoxic substrates for targeted drug delivery and as highly biostable fluorescent markers for cellular tracking. Beyond optical techniques, however, options for noninvasive imaging of nanodiamonds *in vivo* are severely limited. Here, we demonstrate that the Overhauser effect, a proton–electron polarization transfer technique, can enable high-contrast magnetic resonance imaging (MRI) of nanodiamonds in water at room temperature and ultra-low magnetic field. The technique transfers spin polarization from paramagnetic impurities at nanodiamond surfaces to ^1^H spins in the surrounding water solution, creating MRI contrast on-demand. We examine the conditions required for maximum enhancement as well as the ultimate sensitivity of the technique. The ability to perform continuous *in situ* hyperpolarization via the Overhauser mechanism, in combination with the excellent *in vivo* stability of nanodiamond, raises the possibility of performing noninvasive *in vivo* tracking of nanodiamond over indefinitely long periods of time.

## Abstract (web summary)

Hyperpolarized magnetic resonance imaging can enhance imaging contrast by orders of magnitude, but applications are limited by the thermal relaxation of hyperpolarized states. Here, Waddington *et al*. demonstrate the on-demand hyperpolarization of hydrogen spins through the Overhauser effect with nanodiamonds.

Nanoparticles are rapidly emerging as powerful theranostic substrates^1^ for the targeted delivery of vaccines^2^, chemotherapy agents^3^, immunotheraputics^4^, and as a means of tracking tumour distribution on whole-body scales^5^,^6^. Biocompatible nanodiamonds (NDs) are ideal examples, featuring surfaces that are readily functionalized to enable tissue growth and their selective uptake by disease processes^7^,^8^,^9^,^10^. Imaging NDs *in vivo* has been mostly limited to subcellular environments that are optically accessible^11^,^12^. Without imaging modalities beyond optical fluorescence realization of the full theranostic potential of ND to track and investigate complex disease processes, such as metastatic disease, is unlikely.

Magnetic resonance imaging (MRI) is the gold standard for noninvasive high-contrast imaging, but has proven ineffective for directly detecting NDs *in vivo* due to the low abundance and small gyromagnetic ratio of spin-1/2 ^13^C nuclei that comprise the carbon lattice. Dynamic nuclear polarization (DNP) of the ^13^C nuclei at cryogenic temperatures can, in principle, overcome the inherently small nuclear spin polarization of diamond by boosting it some 10,000 times to enable MRI contrast from nanoparticle compounds^13^,^14^,^15^,^16^. Despite these prospects, hyperpolarized nuclei relax to their thermal polarization in a time that, for submicron particles, is short enough to limit the usefulness of the method in an imaging context^13^,^17^.

An alternative approach to tracking ND relies on functionalizing the ND surface with paramagnetic Gd(III) chelates to create complexes for imaging with conventional *T*_1_-weighted MRI^18^. However, this approach faces the challenges of a large background signal, and concern for the long-term toxicity of gadolinium-based compounds^19^.

Here, we demonstrate a different means of imaging and tracking water–ND solutions using Overhauser-enhanced MRI (OMRI)^20^,^21^,^22^,^23^. Operation at ultra-low magnetic field (ULF) enables efficient Overhauser polarization transfer between electronic and nuclear spins in a radio frequency (RF) regime compatible with *in vivo* use. RF pulsing of the electron paramagnetic resonance (EPR) transition between MRI signal acquisitions continually transfers spin polarization from the paramagnetic centres at the surface of ND to ^1^H nuclei in the surrounding water^24^. The presence of ND in the solution thus leads to an enhancement in the ^1^H MRI signal that can readily produce images with contrast sensitive to ND concentrations. The ability to perform *in situ* hyperpolarization overcomes the limitations imposed by short spin relaxation times of smaller particles and enables switchable tracking of ND solutions with no polarization transport losses over indefinite timescales. In addition to producing images to demonstrate this new approach, we investigate the conditions that lead to maximum sensitivity to the presence of ND, presenting data characterizing the efficiency of the Overhauser mechanism as a function of particle concentration and size. These results significantly enhance the theranostic capabilities of non-toxic, biofunctionalized ND, opening the possibility that MRI can be used to monitor and track ND compounds *in vivo*.

## Results

### The Overhauser effect in ND solutions

Various types of ND were used in this study, including high-pressure high-temperature (HPHT), natural (NAT) and detonation (DET) NDs in sizes from 4 to 125 nm. We focus on results obtained from HPHT 18 nm and HPHT 125 nm NDs as typical representatives of the general behaviour observed. An air oxidization process, known to etch the ND surface, produces additional variants of NDs for comparison with the commercially sourced varieties^25^. Aqueous solutions of ND in deionized (DI) water were prepared using high-power probe sonication, with HPHT NDs exhibiting the most stability in solution. HPHT 125 nm solutions show no aggregation over a period of months and a zeta potential of −55 mV (see Methods for further details on ND preparation as well as Supplementary Note 1 and Supplementary Fig. 1a for zeta potential measurements).

**Figure 1:**
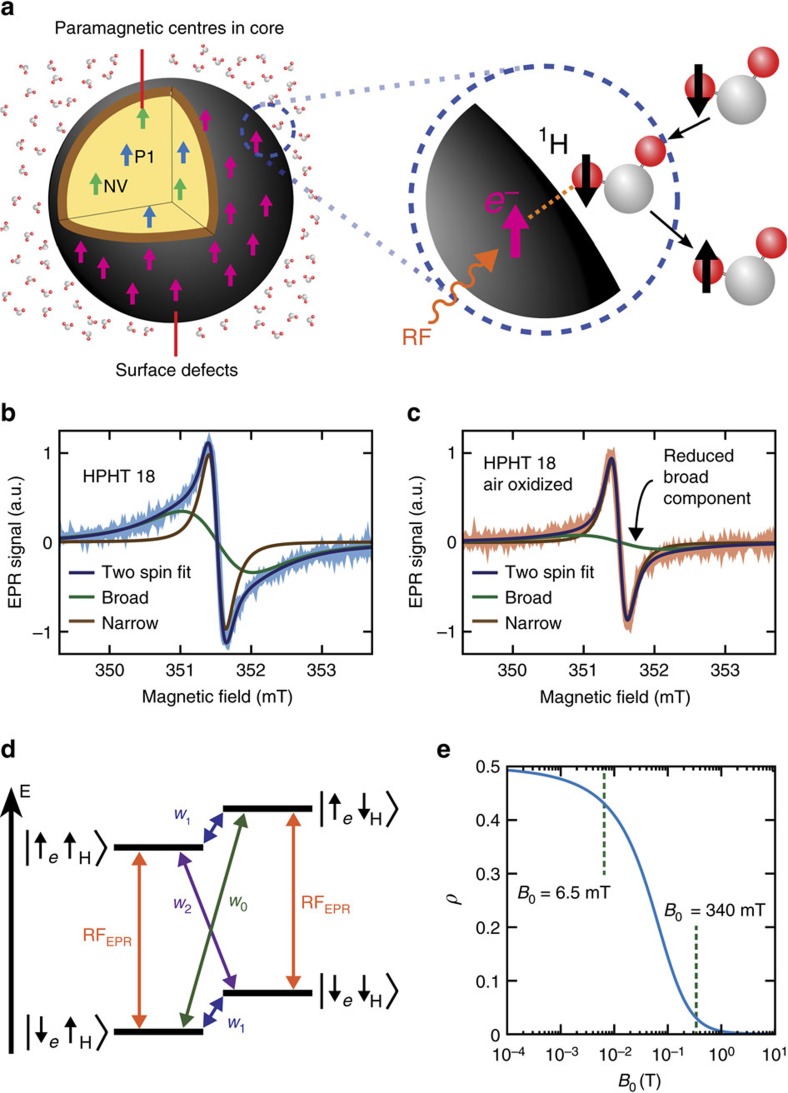
Mechanism of the Overhauser effect in ND solutions. (**a**) Schematic of the Overhauser effect at the ND–water interface. (**b**) X-band EPR spectra of high-pressure high-temperature (HPHT) 18 nm NDs in 100 mg ml^−1^ solutions of DI water (blue). Two-spin model fit (dark blue) is the sum of a broad spin-1/2 component (green) and a narrow spin-1/2 component (brown). (**c**) X-band EPR spectra of air-oxidized HPHT 18 nm NDs in 100 mg ml^−1^ solutions of DI water (orange). Two-spin model fit (dark blue) is the sum of a broad spin-1/2 component (green) and a narrow spin-1/2 component (brown). The broad component is reduced by air oxidation. (**d**) Zeeman split electron and nuclear spin levels in a magnetic field. Zero-quantum (*w*_0_), single-quantum (*w*_1_) and double-quantum transitions (*w*_2_) are shown. If the *w*_2_ transition dominates, when the EPR transition is driven with a RF field, there is a net movement to the 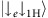 state. (**e**) Coupling factor *ρ* as a function of magnetic field (*B*_0_) for a translational correlation time (*τ*_c_) of 430 ns.

The basis for detecting and imaging ND in solution is shown in Fig. 1a. Image contrast arises from the Overhauser effect, which as a starting point requires a reservoir of partially polarized electron spins^26^. Driving these electrons with a resonant AC magnetic field transfers spin polarization to the interacting ^1^H nuclei in the surrounding solution^27^,^28^. NDs provide such a reservoir in the form of paramagnetic impurities such as nitrogen vacancy centres, substitutional nitrogen (P1) centres and unpaired electrons at the nanoparticle surface^29^,^30^. We first characterize our NDs using EPR spectroscopy, determining their impurity content and suitability for Overhauser imaging.

The EPR spectra of our HPHT 18 nm ND solution is shown in Fig. 1b and is fit by a two-component spin-1/2 model comprising a broad (1.2 mT) component and a narrow (0.2 mT) component (solid lines in figure)^31^. Air oxidation of NDs reduces the amplitude of the broad component in the spectra, as shown in Fig. 1c, presumably by removing the paramagnetic centres at the surface. Our results are consistent with previous studies suggesting that the broad component is due to disordered dangling bonds at the surface of the ND with the narrow component arising from lattice defects in the crystalline core^32^. Other types of ND studied here demonstrate similar spectral components (see Supplementary Fig. 2 for further EPR data).

Having established that ND provides a paramagnetic reservoir suitable for the Overhauser effect, we turn now to address the additional conditions that must be satisfied to enable imaging. In Fig. 1d we show the energy level diagram of a system comprising an electron coupled to a ^1^H nucleus in an external magnetic field. When the EPR transition is pumped, the relative sizes of the cross relaxation transitions *w*_0_ and *w*_2_ will cause nuclei to accumulate in spin up or down states. This accumulation gives a nuclear enhancement 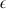, defined as the ensemble average of the *z*-component of the nuclear magnetization *M*_*z*_ over the nuclear magnetization under thermal equlibrium conditions *M*_0_, that is, 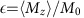. The enhancement generated by the Overhauser mechanism is a function of four parameters^33^:


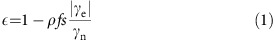


where *ρ* is the coupling factor between electron and nuclear spins, *f* the leakage factor, *s* the saturation factor and *γ*_e_ and *γ*_n_ are the electron and nuclear gyromagnetic ratios. Addressing first the coupling factor, *ρ*, we note that when there is dipolar coupling but no hyperfine contact interaction between spins, *ρ* takes a positive value determined by the correlation time of the two spins, diffusion coefficients and the EPR frequency. Relatively long correlation times are expected at ND surfaces due to the formation of a nanophase of water with 1 nm thickness at the ND–water interface^34^. Assuming free diffusion of water at a distance of 1 nm from the ND surface, we follow refs 35, 36, 37, 38 and plot the field dependence of *ρ* for a calculated correlation time of 430 ns, as shown in Fig. 1e (see Supplementary Note 2 for details of calculation). Not surprisingly, given the long correlation time between spins, increasing the magnetic field above a few milli-Tesla rapidly suppresses the mutual flip–flip of dipolar coupled electron and nuclear spins and thus the nuclear enhancement possible via the Overhauser effect. Our choice of magnetic field for Overhauser imaging is thus constrained to the ULF regime, where serendipitously the frequency of the EPR field produces minimal heating from dielectric loss associated with water at 20 °C (ref. 39). Figure 1e also explains why previous DNP studies of ND solutions at 340 mT did not show Overhauser enhancement of freely diffusing water molecules in the bulk solution^40^. Instead, they showed solid effect DNP of ^1^H nuclei adsorbed to the ND surface.

**Figure 2:**
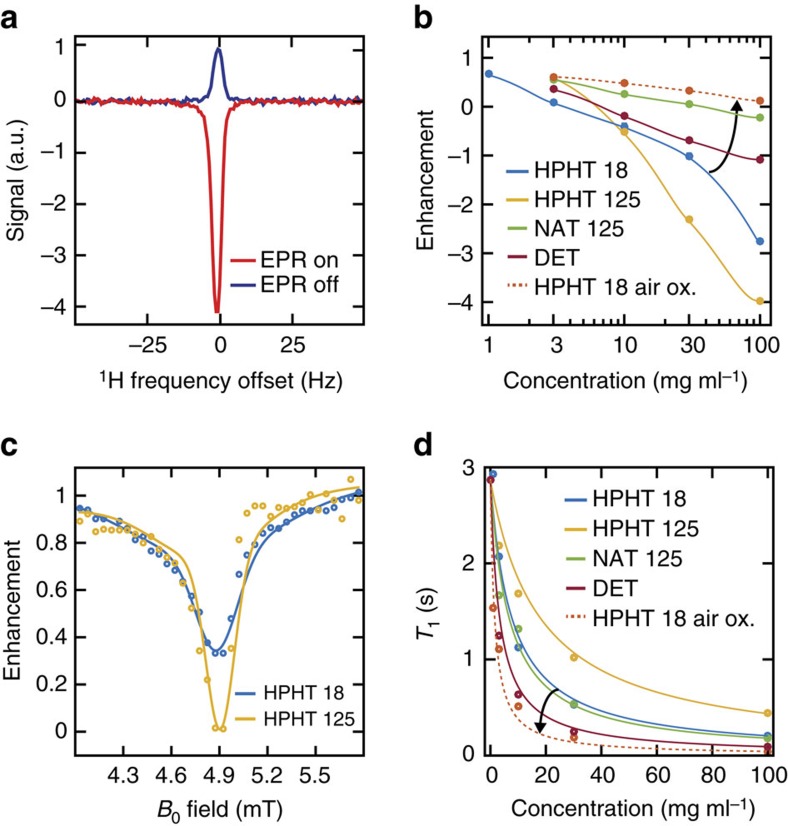
Overhauser effect in ND solutions. (**a**) NMR spectra acquired at 276 kHz demonstrating Overhauser enhancement of ^1^H polarization in an HPHT 125 nm 100 mg ml^−1^ ND solution. The hyperpolarized ^1^H spectrum (red) is enhanced by −4.0 over the thermal ^1^H spectrum (blue). The enhanced spectrum was acquired after the EPR transition had been driven for 1.5 s with 49 W of RF power at 190 MHz. (**b**) ^1^H saturation enhancement versus concentration for ND solutions at 6.5 mT, with EPR saturation of 49 W at 190 MHz. The Overhauser effect was observed for HPHT (blue—18 nm; yellow—125 nm), NAT 125 nm (green), DET (red) and air-oxidized HPHT 18 nm NDs (orange). Lines are included as a guide to the eye. Arrow indicates the change in enhancement after air oxidation. (**c**) ^1^H enhancement versus *B*_0_ with EPR pumping at a constant frequency of 140 MHz with 24 W of power. ^1^H NMR detection was performed on resonance. Aqueous solutions of HPHT 18 nm (blue) and HPHT 125 nm (yellow) at 50 mg ml^−1^ concentration were used. Solid lines are included as a guide to the eye. (**d**) *T*_1_ relaxation times of ND solutions at 6.5 mT. Lines are a fit to the concentration-dependent relaxivity equation. The fit error on individual *T*_1_ measurements is smaller than the marker size. Arrow indicates the change in relaxivity after air oxidation. The *T*_1_ relaxivity coefficients are 4.5±0.2 × 10^−2^ ml s^−1^ mg^−1^ for HPHT 18 nm (blue), 1.9±0.2 × 10^−2^ ml s^−1^ mg^−1^ for HPHT 125 nm (yellow), 5.2±0.2 × 10^−2^ ml s^−1^ mg^−1^ for NAT 125 nm (green), 1.0±0.1 × 10^−1^ ml s^−1^ mg^−1^ for DET (red) and 2.3±0.2 × 10^−1^ ml s^−1^ mg^−1^ for air-oxidized HPHT 18 nm (orange).

To demonstrate that NDs can be detected via the Overhauser effect at ULF, we set *B*_0_=6.5 mT and apply an RF magnetic field at the EPR frequency of 190 MHz to an HPHT 125 nm, 100 mg ml^−1^ sample. The ^1^H signal from the water surrounding the ND is then detected through standard inductive nuclear magnetic resonance (NMR) techniques after the ^1^H system has reached equilibrium (see Methods). Under these conditions, we observe an enhancement of −4.0 in the ^1^H spin polarization when EPR power is applied, as shown in Fig. 2a.

Examining the enhancement produced by different types of ND, Fig. 2b shows the sensitivity of the Overhauser technique to nanoparticle concentration. We draw attention to the data for the HPHT 18 nm NDs, which indicates that at concentrations of 1 mg ml^−1^, a 33% change in ^1^H polarization of the solution can be observed. Natural NDs produce a small enhancement relative to HPHT NDs, probably due to a relatively low concentration of paramagnetic defects, as seen in their EPR spectra (see Supplementary Fig. 2 for spectra).

Having demonstrated that Overhauser enhancement is possible with ND, we return to equation (1) to further consider the conditions needed for optimal imaging. The saturation factor *s* describes the proportion of the EPR linewidth that is driven and takes a maximum value of 1 when the electron transitions are completely saturated at high RF power. To measure the EPR linewidth at ULF, we sweep *B*_0_ while driving electron transitions at 140 MHz. As shown in Fig. 2c, HPHT 18 nm and HPHT 125 nm solutions show a linewidth for the enhancement of ∼0.3 mT at a frequency consistent with the gyromagnetic ratio of a free electron. This result indicates that the paramagnetic centres responsible for the Overhauser effect can be fully saturated with a resonant AC magnetic field of magnitude 1 mT, which is easily achieved in our spectroscopic probe^41^. Accordingly, we observe that, for EPR powers above 30 W, the Overhauser enhancement saturates, (shown in Supplementary Fig. 1b), and the maximal saturation factor is reached.

The remaining parameter in equation (1) is the leakage factor *f*, which describes how effectively electrons relax the nuclear spin environment, taking a maximum value of 1 when all nuclear spin relaxation is via the paramagnetic solute. The leakage factor of a given solution can be calculated from:


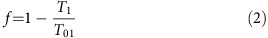


where *T*_1_ is the spin–lattice relaxation time of ^1^H spins in ND solution, shown in Fig. 2d, and *T*_01_ is the spin–lattice relaxation time of the undoped solvent^26^. We note that ND solutions with shorter *T*_1_, and hence larger *f*, do not necessarily give a higher Overhauser enhancement as equation (1) would predict. For example, NAT 125 nm NDs show a *T*_1_ relaxivity more than double that of HPHT 125 nm NDs, despite showing a much smaller enhancement in Fig. 2b. Presumably, *ρ* is suppressed in the quasistatic nanophase by slow diffusion of water molecules and the increased paramagnetic nuclear relaxation rate^40^. ^1^H nuclei ‘trapped' in the nanophase will experience rapid spin–lattice relaxation, giving the *f* we observe and an overall enhancement that depends on the specifics of each ND surface. A detailed understanding of dynamics in the nanophase compared to freely diffusing bulk water is thus crucial to calculation of the factors in equation (1) (see Supplementary Note 3 for further discussion).

Solutions prepared with air-oxidized ND consistently exhibit reduced enhancements and higher *T*_1_ relaxivity, as shown for 18 nm HPHT air-oxidized NDs in Fig. 2b,d. The increased nuclear spin–lattice relaxation rate will contribute to a reduction in Overhauser enhancement and we speculate that the enhancement is further reduced due to a lower concentration of paramagnetic centres after removal of surface impurities by air oxidation.

### Overhauser-enhanced MRI with ND

**Figure 3:**
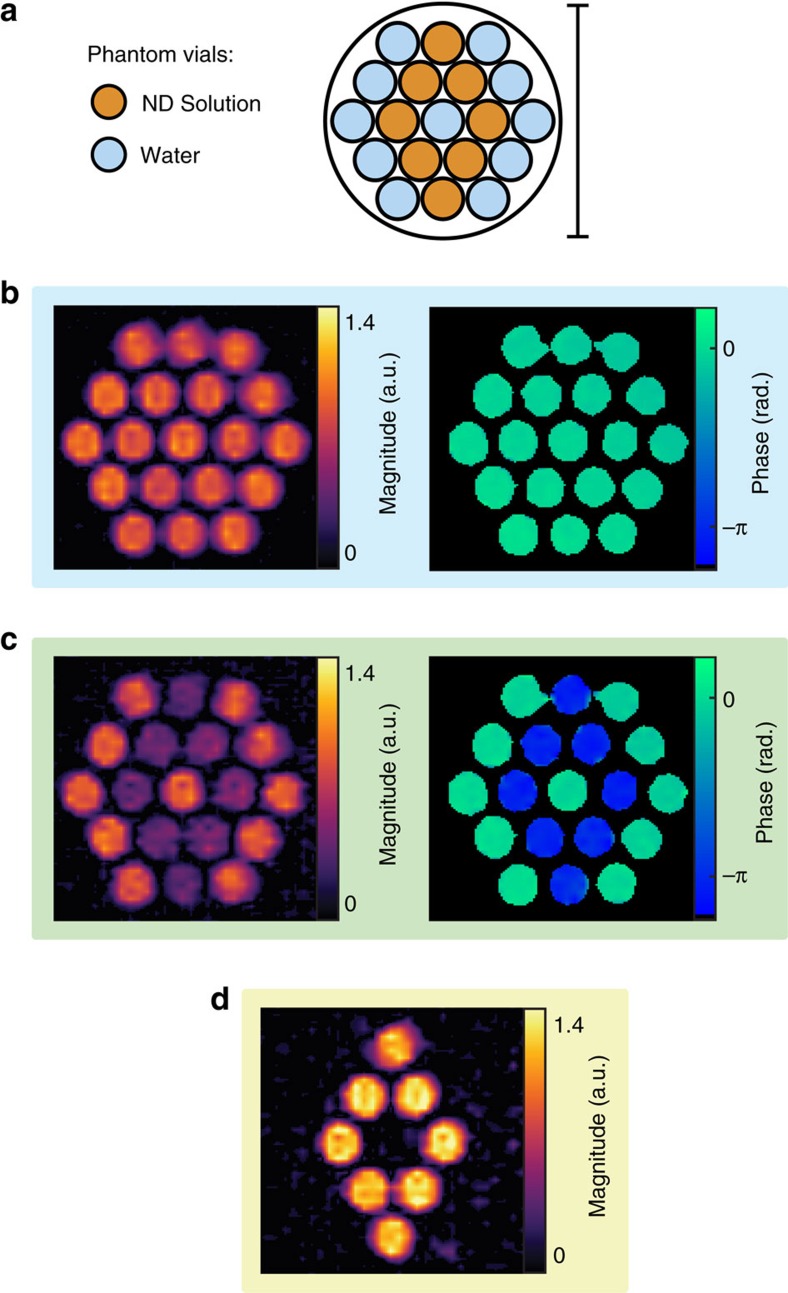
ND imaging with Overhauser-enhanced MRI. (**a**) Imaging phantom. Vials of DI water (blue) and vials of HPHT 125 nm ND at 100 mg ml^−1^ (orange) were arranged in the pattern shown. Scale bar is 30 mm in length. (**b**) Standard bSSFP MRI of the phantom shown in panel **a**. Magnitude colourscale is normalized such that water has a magnitude of 1. Phase image is masked (black) in regions where the signal magnitude is less than than five times the root mean square value of the background signal. (**c**) OMRI bSSFP image of the same phantom. The Overhauser effect generates contrast between ND solution and water. The phase of the acquired signal is inverted in the ND solution. Magnitude colourscale is the same as that used in **b**. Phase image is masked (black) with the same mask applied to the phase image in **b**. (**d**) The difference of MRI and OMRI acquisitions. The background signal is suppressed, showing signal only where ND is present. Magnitude colourscale is the same as that used in **b**.

With conditions that lead to a significant Overhauser enhancement now established, we demonstrate this approach as the basis for detecting ND solutions using ultra-low-field MRI. Imaging is performed using a custom proton–electron, double resonant probe in an open-access, low-field, human MRI scanner operating at a *B*_0_ of 6.5 mT (ref. 42). To display the MRI contrast possible between a ND solution and water we make use of the phantom illustrated in Fig. 3a, which consists of glass vials filled with 500 μl of either DI water or aqueous solutions of HPHT 125 nm ND at 100 mg ml^−1^ and is organized in a diamond-shaped pattern.

MRI at ultra-low field of the phantom was performed using a high-efficiency balanced steady-state free precession (bSSFP) MRI sequence in which 1/3 of the imaging time is spent acquiring signal (see Methods for details)^43^. Although good spatial resolution is achieved, no discernible contrast is evident between ND solution vials and water vials, as shown in Fig. 3b. This is not surprising given that contrast using the bSSFP sequence is produced via ^1^H concentration weighted by the ratio *T*_2_/*T*_1_, which is approximately equal for all vials in the phantom (see Fig. 2d for *T*_1_ and Supplementary Fig. 1c for *T*_2_). We note that obtaining relaxation contrast with bSSFP at ULF is usually challenging, as when *B*_0_→0, it is a general result that *T*_2_/*T*_1_→1 (ref. 44).

The phantom was then imaged with an OMRI bSSFP sequence, as shown in Fig. 3c. The OMRI bSSFP sequence is equivalent to the regular bSSFP sequence, except the EPR transition of the ND solution is driven during the phase encode period^24^. The maximum time period without Overhauser saturation in our OMRI bSSFP sequence is 28 ms. As this is much shorter than the *T*_1_ and *T*_2_ of the ND solution, the polarization approaches a steady state during OMRI bSSFP, ensuring that hyperpolarized signal is continually present for acquisition. The appearance of water vials in the OMRI bSSFP image is unchanged from the regular bSSFP image. However, the ND solutions demonstrate significant relative contrast, with a change in magnitude and inversion of signal phase, as a result of the negative enhancement from the Overhauser effect. The switchable nature of the Overhauser contrast allows us to take the difference of the signal in MRI and OMRI images to generate the image in Fig. 3d. Such a difference image suppresses the background signal, clearly showing the spatial distribution of NDs.

**Figure 4:**
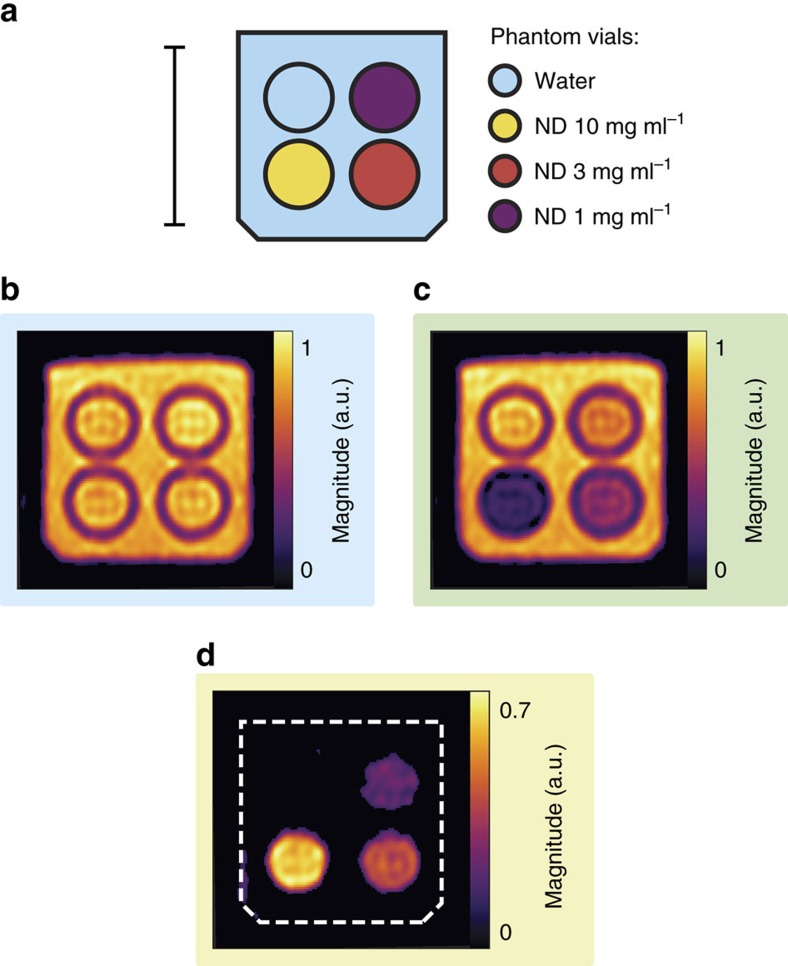
Sensitivity of ND Imaging. (**a**) Phantom schematic. A vial of DI water (blue) and vials of HPHT 18 nm ND at concentrations of 10 mg ml^−1^ (yellow), 3 mg ml^−1^ (red) and 1 mg ml^−1^ (purple) were mounted in the phantom as shown. The surrounding volume was then filled with water (blue). Scale bar is 20 mm in length. (**b**) Standard bSSFP MRI of the phantom shown in panel **a**. Colourscale is normalized such that water has a magnitude of 1. (**c**) OMRI bSSFP image of the same phantom. The phase of the MRI signal is uniformly positive across the image. Colourscale is the same as that used in panel **b**. (**d**) The difference of MRI and OMRI acquisitions. The water signal is suppressed and all ND vials clearly visible. Colourscale is the same as that used in panel **b**.

Having demonstrated ND imaging with OMRI, we now consider the sensitivity of the technique in our current Overhauser setup. We calculate the signal-to-noise ratio (SNR) as the magnitude of the MRI signal in a region of interest divided by the root mean square value of the background signal. In Fig. 4a we show the schematic of a phantom containing vials with various concentrations of HPHT 18 nm ND in a container of water. This phantom is imaged with bSSFP, as shown in Fig. 4b. The vials in this image have an SNR of 43, with the glass vial walls clearly outlining their positions. Next, we define the contrast-to-noise ratio (CNR) as the difference in signal between MRI and OMRI scans in a region of interest divided by the root mean square value of the background signal. Taking the subsequent Overhauser scan, shown in Fig. 4c and resulting difference image, shown in Fig. 4d, clearly shows the presence of ND at concentrations of 10, 3 and 1 mg ml^−1^ with CNR values of 27, 18 and 9, respectively.

Images of the 1 mg ml^−1^ vial in Fig. 4 were acquired with 23 μg of ND per pixel, or a particle molar sensitivity of 150 nM for 18 nm particles. We note that this particle mass sensitivity is equivalent to that reported for other hyperpolarized MRI particle imaging modalities^17^.

## Discussion

NDs are non-toxic at high concentrations and resist *in vivo* degradation for periods of over a month^45^. Thus, the results presented here illustrate the potential of ND OMRI as a practical methodology for long-term biological imaging, providing new types of contrast and functionality. All imaging was performed on systems designed for *in vivo* OMRI with RF powers acceptable for use *in vivo*^46^, raising the possibility of biological applications. In particular, NDs may be of diagnostic use for diseased organs where nanoparticle accumulation can be an effective marker of pathology, such as the brain^47^, liver^48^ and lymph nodes^2^. Current diagnostic methodologies, using the 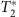 properties of iron oxide nanoparticles^49^, suffer from the long biodistribution times of nanoparticles as the precomparison scan is taken before nanoparticle administration. In the resulting interval before a postcomparison scan various types of biological noise are introduced that make difference imaging infeasible^50^. The ability to perform interleaved MRI and OMRI scans with ND could overcome this limitation.

We have demonstrated sensitivity to ND at concentrations as low as 1 mg ml^−1^. An upgraded version of our scanner with higher strength imaging gradients will enable slice selection without compromise to the bSSFP acquisition protocol. Hence, we now anticipate sensitivity changes from the implementation of slice selection. The SNR in a slice-selected image is given by SNR=*κV*_voxel_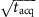, where *V*_voxel_ is the volume of a voxel, *t*_acq_ is the total acquisition time and *κ* is a constant that depends on the magnetic field strength, hardware sensitivity, imaging sequence and acquisition parameters as well as the composition and spin–relaxation properties of the material being imaged^51^. Hence, based on the measurements in Fig. 4, for the same acquistion time and slice selection in a 5 mm slab with 1 mm × 1 mm pixel size, we calculate that HPHT 18 nm ND at 1 mg ml^−1^ will be on the threshold of detectability with a CNR of 2. For higher nanoparticle concentrations, imaging times could be significantly accelerated. For example, HPHT 18 nm ND at 10 mg ml^−1^ will have a CNR of 2, with 5 mm^3^ voxels and a total MRI and OMRI acquisition time of 2.5 min.

The long-term clearance of nanoparticles is of interest for assessing biocompatibility. Studying the retention of NDs in the liver at present requires organ harvesting, which limits long-term studies^45^,^52^. We estimate that for NDs to be present at 1 mg ml^−1^ in a 3 ml mouse liver^53^ would require injection of 5 mg of ND into a 20 g mouse, assuming 60% accumulation in the liver^53^,^54^. This dose is significant as it is a factor of 30 lower than that used in a recent *in vivo* demonstration of hyperpolarized silicon microparticle imaging^17^. Nanoparticle accumulation could be noninvasively imaged at this concentration with 5 mm^3^ voxels in the 3,000 mm^3^ liver. This would provide a long-term probe of the fate of nanoparticles in the liver, with significant scope for increasing the voxel size or acquisition time if increased sensitivity is required.

Given that nanoparticles about 25 nm in size are known to preferentially accumulate only in healthy lymphatic tissue^2^, the ability to detect and image ND with OMRI may also enable isolation of disease in swollen lymph glands, avoiding the need for biopsy^55^,^56^,^57^. Such a technique could prove useful for the diagnosis of lymph node tumours, which is vital to the treatment of metastatic prostate cancer^6^.

The ^1^H enhancement we observe with the Overhauser effect is approximately two orders of magnitude larger, when accounting for ND concentration, than seen with solid effect hyperpolarization of water molecules adsorbed to ND surfaces^40^. Further, there is potential to increase the Overhauser enhancement towards the theoretical maximum of −330 (ref. 26), over 80 times larger than seen here, by modification of the ND surface. Tailored NDs with impurities selected to remove alternate spin–lattice relaxation mechanisms could be surface treated to increase diffusion of water at the nanosolid–liquid interface^58^,^59^, maximizing the coupling and leakage factors in equation (1). Likely, other nanoparticles that display the Overhauser effect in solution also exist. However, identification of these nanoparticles is nontrivial because of significant variation in the surface defects and the hydrophilicity of nanoparticles.

As Overhauser contrast arises via interactions at the nanoparticle surface, we recognize that surface functionalization for targeted molecular imaging must complement the observed enhancement. In this way, therapeutic agents attached to the surface could suppress the Overhauser effect by increasing the distance between radicals at their surface and free water, leaving them ‘dark' in OMRI scans^60^. After targeted drug release, the Overhauser effect could return to normal, showing up ‘bright' with OMRI and enabling effective tracking of the site of drug delivery. The dependence of Overhauser enhancement on diffusion may also allow the technique to be used as a probe of localized hydration dynamics^35^. The OMRI approach may also enable the hyperpolarization of fluids flowing across the surface of diamond nanostructures^61^,^62^,^63^.

In conclusion, we have used recent advances in ULF MRI to extend the usefulness of OMRI to nanoparticle imaging. The ability to noninvasively image biocompatible NDs with switchable contrast at biologically relevant concentrations is promising for a range of diagnostic applications. Switchable contrast allows suppression of the background signal present in other *T*_1_- and *T*_2_-based nanoparticle MRI modalities^18^,^64^,^65^. Furthermore, the long-term biological stability of NDs *in vivo*, as well as the unlimited repeatability of the hyperpolarization sequence, raises the possibility of imaging metabolic processes over dramatically longer timescales than is possible with *ex situ* hyperpolarization techniques^13^,^17^.

## Methods

### ND solution preparation

NDs used in this study were sourced from Microdiamant (Switzerland). ND types used were: monocrystalline, synthetic HPHT NDs in 18 nm (0–30 nm, median diameter 18 nm) and 125 nm (0–250 nm, median diameter 125 nm) sizes; monocrystalline, 125 nm NAT NDs (0–250 nm, median diameter 125 nm); and polycrystalline DET ND (cluster size 250–1,000 nm, median 500 nm; individual particle size 4–8 nm). Size specifications were provided by the manufacturer. Air-oxidized NDs were prepared by placing them in a furnace at standard pressure for 1 h at 550 °C after an initial temperature ramp^25^. ND samples were mixed with DI water and sonicated with a Branson probe sonicator at 120 W and 50% duty cycle for 40 min to disaggregate ND clusters.

Particle size and zeta potential measurements were performed on ND solutions in a Beckman Coulter Delsa Nano C Particle Analyzer. Particle size measurements confirmed that monocrystalline NDs were well dispersed in water after sonication. Particle sizes of monocrystalline NDs were found to be consistent with manufacturer specifications. DET NDs still displayed some clustering and inconsistent particle size in solution after probe sonication.

### EPR measurements

CW EPR measurements were performed on 100 mg ml^−1^ ND samples in a Bruker ElexSys E500 EPR system. Modulation frequency was 100 kHz at an amplitude of 0.1 G and incident microwave power of 0.6725, mW. Sample volumes in the cavity were kept consistent to allow comparison of relative peak heights. Individual EPR components were simulated in EasySpin^31^ and a least-squares analysis was used to find the best fit to the data while varying *g*-factor, linewidth and amplitude.

### Spectroscopic measurements at 6.5 mT

The ^1^H enhancement of ND solutions was measured by saturating the EPR transition at 190 MHz for a period of 5*T*_1_ using 31 W of RF power before a standard NMR FID acquisition at 276 kHz. The enhancement is given as the ratio of the magnitude of the hyperpolarized FID to the magnitude an FID taken at thermal equilibrium with no EPR power. A high filling factor Alderman–Grant resonator was used for EPR with a solenoid-producing orthogonal *B*_1_ used for NMR detection. *T*_1_ relaxation times were measured using a conventional inversion recovery acquisition and fit with a least-squares analysis.

### DNP linewidth measurements

The linewidth of the Overhauser effect was studied by measuring the ^1^H enhancement at various magnetic field strengths while driving the EPR resonator at a frequency of 140 MHz and 25 W. The EPR frequency was lowered from 190 MHz to capture the enhancement profile either side of the peak without exceeding the maximum field accessible in our ULF magnet. ^1^H enhancement was measured as the magnetic field was stepped between 4 and 7 mT. NMR detection was performed at the ^1^H resonance for any given field strength, with a low *Q* solenoidal coil.

### Overhauser-enhanced MRI

Imaging was performed at 6.5 mT in our ultra-low-field MRI scanner^42^ using a bSSFP OMRI sequence at room temperature^24^. The homebuilt imaging probe consists of an Alderman–Grant resonator (EPR: 190 MHz) and a solenoid (^1^H: 276 kHz). The EPR resonator was pulsed on during the phase encode steps, with 69 W delivered to the EPR resonator at a duty cycle of 52%. Gradient strength was a maximum of 1 mT m^−1^ (see Supplementary Note 4 and Supplementary Fig. 3 for further imaging sequence and probe details). Images in Figs 3 and 4 were acquired with a 256 × 40 matrix size and cropped. Data were acquired in two dimensions with a pixel size of 1.0 mm × 0.76 mm over a 30 mm × 30 mm field of view and interpolated by zero filling and Gaussian filtering in *k*-space to give 0.25 mm × 0.19 mm pixels. The phantom thickness was 20 and 30 mm in Figs 3 and 4, respectively. The standard MRI images were acquired with 200 averages (11 min 24 s). The OMRI images in Figs 3c and 4c were acquired with 80 averages (4 min 14 s) and 200 averages (11 min 24 s), respectively. Image magnitude was scaled for an accurate comparison between scans with different numbers of averages. Pixels in Fig. 4 with a magnitude less than five times the root mean square value of the background signal were thresholded to zero.

### Data availability

The authors declare that the data supporting the findings of this study are available within the article and its Supplementary Information. Raw data is available from the corresponding author on request.

## Additional information

**How to cite this article:** Waddington, D. E. J. *et al*. Nanodiamond-enhanced MRI via *in situ* hyperpolarization. *Nat. Commun.* **8,** 15118 doi: 10.1038/ncomms15118 (2017).

**Publisher's note**: Springer Nature remains neutral with regard to jurisdictional claims in published maps and institutional affiliations.

## Supplementary Material

Supplementary Information Supplementary Figures, Supplementary Notes and Supplementary References.

## References

[b1] Min Y., Caster J. M., Eblan M. J. & Wang A. Z. Clinical translation of nanomedicine. Chem. Rev. 115, 11147–11190 (2015). 26088284 10.1021/acs.chemrev.5b00116 PMC4607605

[b2] Reddy S. T. . Exploiting lymphatic transport and complement activation in nanoparticle vaccines. Nat. Biotechnol. 25, 1159–1164 (2007). 17873867 10.1038/nbt1332

[b3] Gaur S. . Pharmacodynamic and pharmacogenomic study of the nanoparticle conjugate of camptothecin CRLX101 for the treatment of cancer. Nanomed. Nanotechnol. 10, 1477–1486 (2014). 10.1016/j.nano.2014.04.003 24768630

[b4] Almeida J. P. M., Figueroa E. R. & Drezek R. A. Gold nanoparticle mediated cancer immunotherapy. Nanomed. Nanotechnol. 10, 503–514 (2014). 10.1016/j.nano.2013.09.011 PMC3966952 24103304

[b5] Liu Y. . Theranostic near-infrared fluorescent nanoplatform for imaging and systemic siRNA delivery to metastatic anaplastic thyroid cancer. Proc. Natl Acad. Sci. USA 113, 7750–7755 (2016). 27342857 10.1073/pnas.1605841113 PMC4948349

[b6] Harisinghani M. G. . Noninvasive detection of clinically occult lymph-node metastases in prostate cancer. N. Engl. J. Med. 348, 2491–2499 (2003). 12815134 10.1056/NEJMoa022749

[b7] Chow E. K. . Nanodiamond therapeutic delivery agents mediate enhanced chemoresistant tumor treatment. Sci. Transl. Med. 3, 73ra21 (2011). 10.1126/scitranslmed.3001713 21389265

[b8] Xi G. . Convection-enhanced delivery of nanodiamond drug delivery platforms for intracranial tumor treatment. Nanomed. Nanotechnol. 10, 381–391 (2014). 10.1016/j.nano.2013.07.013 23916888

[b9] Thalhammer A., Edgington R. J., Cingolani L. A., Schoepfer R. & Jackman R. B. The use of nanodiamond monolayer coatings to promote the formation of functional neuronal networks. Biomaterials 31, 2097–2104 (2010). 20035997 10.1016/j.biomaterials.2009.11.109

[b10] Zhang Q. . Fluorescent PLLA-nanodiamond composites for bone tissue engineering. Biomaterials 32, 87–94 (2011). 20869765 10.1016/j.biomaterials.2010.08.090

[b11] Schrand A. M. . Are diamond nanoparticles cytotoxic? J. Phys. Chem. B 111, 2–7 (2007). 17201422 10.1021/jp066387v

[b12] McGuinness L. P. . Quantum measurement and orientation tracking of fluorescent nanodiamonds inside living cells. Nat. Nanotechnol. 6, 358–363 (2011). 21552253 10.1038/nnano.2011.64

[b13] Rej E., Gaebel T., Boele T., Waddington D. E. J. & Reilly D. J. Hyperpolarized nanodiamond with long spin–relaxation times. Nat. Commun. 6, 8459 (2015). 26450570 10.1038/ncomms9459 PMC4633625

[b14] Casabianca L. B., Shames A. I., Panich A. M., Shenderova O. & Frydman L. Factors affecting DNP NMR in polycrystalline diamond samples. J. Phys. Chem. C 115, 19041–19048 (2011).

[b15] Dutta P., Martinez G. V. & Gillies R. J. Nanodiamond as a new hyperpolarizing agent and its ^13^C MRS. J. Phys. Chem. Lett. 5, 597–600 (2014). 26276615 10.1021/jz402659t

[b16] Bretschneider C. O. . On the potential of dynamic nuclear polarization enhanced diamonds in solid-state and dissolution ^13^C NMR spectroscopy. Chem. Phys. Chem. 17, 2691–2701 (2016). 27416769 10.1002/cphc.201600301

[b17] Cassidy M. C., Chan H. R., Ross B. D., Bhattacharya P. K. & Marcus C. M. *In vivo* magnetic resonance imaging of hyperpolarized silicon particles. Nat. Nanotechnol. 8, 363–368 (2013). 23644571 10.1038/nnano.2013.65

[b18] Manus L. M. . Gd(III)-nanodiamond conjugates for MRI contrast enhancement. Nano Lett. 10, 484–489 (2010). 20038088 10.1021/nl903264h PMC2829273

[b19] McDonald R. J. . Intracranial gadolinium deposition after contrast-enhanced MR imaging. Radiology 275, 772–782 (2015). 25742194 10.1148/radiol.15150025

[b20] Lurie D. J., Li H., Petryakov S. & Zweier J. L. Development of a PEDRI free-radical imager using a 0.38 T clinical MRI system. Magn. Reson. Med. 47, 181–186 (2002). 11754457 10.1002/mrm.10029

[b21] Golman K., Leunbach I., Petersson J. S., Holz D. & Overweg J. Overhauser-enhanced MRI. Acad. Radiol. 9, (Suppl 1): S104–S108 (2002). 12019840 10.1016/s1076-6332(03)80411-7

[b22] Koonjoo N. . *In vivo* Overhauser-enhanced MRI of proteolytic activity. Contrast Media Mol. Imag. 9, 363–371 (2014). 10.1002/cmmi.1586 24729587

[b23] Ichikawa K. & Yasukawa K. Imaging *in vivo* redox status in high spatial resolution with OMRI. Free Radical Res. 46, 1004–1010 (2012). 22375816 10.3109/10715762.2012.670874

[b24] Sarracanie M., Armstrong B. D., Stockmann J. & Rosen M. S. High speed 3D Overhauser-enhanced MRI using combined b-SSFP and compressed sensing. Magn. Reson. Med. 71, 735–745 (2013). 10.1002/mrm.24705 23475813

[b25] Gaebel T. . Size-reduction of nanodiamonds via air oxidation. Diam. Rel. Mater. 21, 28–32 (2012).

[b26] Ravera E., Luchinat C. & Parigi G. Basic facts and perspectives of Overhauser DNP NMR. J. Magn. Reson. 264, 78–87 (2016). 26920833 10.1016/j.jmr.2015.12.013

[b27] Clarkson R. B. . Electron paramagnetic resonance and dynamic nuclear polarization of char suspensions: surface science and oximetry. Phys. Med. Biol. 43, 1907–1920 (1998). 9703054 10.1088/0031-9155/43/7/012

[b28] Ardenkjaer-Larsen J. H. . EPR and DNP properties of certain novel single electron contrast agents intended for oximetric imaging. J. Magn. Reson. 133, MN981438 (1998). 10.1006/jmre.1998.1438 9654463

[b29] Panich A. M., Sergeev N. A., Shames A. I., Osipov V. Y. & Boudou J. P. Size dependence of ^13^C nuclear spin–lattice relaxation in micro- and nanodiamonds. J. Phys. Condens. Matter 27, 72203 (2015). 10.1088/0953-8984/27/7/072203 25646270

[b30] Cui J.-F., Fang X.-W. & Schmidt-Rohr K. Quantification of C=C and C=O surface carbons in detonation nanodiamond by NMR. J. Phys. Chem. C 118, 9621–9627 (2014).

[b31] Stoll S. & Schweiger A. EasySpin, a comprehensive software package for spectral simulation and analysis in EPR. J. Magn. Reson. 178, 42–55 (2006). 16188474 10.1016/j.jmr.2005.08.013

[b32] Yavkin B. V., Mamin G. V., Gafurov M. R. & Orlinskii S. B. Size-dependent concentration of N^0^ paramagnetic centres in HPHT nanodiamonds. Magn. Reson. Solids 17, 15101 (2015).

[b33] Günther U. L. Dynamic nuclear hyperpolarization in liquids. Top. Curr. Chem. 335, 23–69 (2011). 10.1007/128_2011_229 22025060

[b34] Korobov M. V., Avramenko N. V., Bogachev A. G., Rozhkova N. N. & Osawa E. Nanophase of water in nano-diamond gel. J. Phys. Chem. C 111, 7330–7334 (2007).

[b35] Armstrong B. D. & Han S. Overhauser dynamic nuclear polarization to study local water dynamics. J. Am. Chem. Soc. 131, 4641–4647 (2009). 19290661 10.1021/ja809259q

[b36] Tóth É., Helm L. & Merbach A. E. Relaxivity of MRI contrast agents. Top. Curr. Chem. 221, 61–101 (2002).

[b37] Hwang L.-P. & Freed J. H. Dynamic effects of pair correlation functions on spin relaxation by translational diffusion in liquids. J. Chem. Phys. 63, 4017–4025 (1975).

[b38] Freed J. Dynamic effects of pair correlation functions on spin relaxation by translational diffusion in liquids. II. Finite jumps and independent T1 processes. J. Chem. Phys. 68, 4034–4037 (1978).

[b39] Gabriel C., Gabriel S., Grant E. H., Halstead B. S. J. & Mingos D. M. P. Dielectric parameters relevant to microwave dielectric heating. Chem. Soc. Rev. 27, 213–223 (1998).

[b40] Rej E., Gaebel T., Waddington D. E. J. & Reilly D. J. Hyperpolarized nanodiamond surfaces. J. Am. Chem. Soc. 139, 193–199 (2017). 28009158 10.1021/jacs.6b09293

[b41] Mispelter J., Mikaela L. & Briguet A. NMR Probeheads for Biophysical and Biomedical Experiments Imperial College Press (2006).

[b42] Sarracanie M. . Low-cost high-performance MRI. Sci. Rep. 5, 15177 (2015). 26469756 10.1038/srep15177 PMC4606787

[b43] Scheffler K. & Lehnhardt S. Principles and applications of balanced SSFP techniques. Eur. Radiol. 13, 2409–2418 (2003). 12928954 10.1007/s00330-003-1957-x

[b44] Bottomley P. A., Foster T. H., Argersinger R. E. & Pfeifer L. M. A review of normal tissue hydrogen NMR relaxation times and relaxation mechanisms from 1–100 MHz: dependence on tissue type, NMR frequency, temperature, species, excision, and age. Med. Phys. 11, 425–448 (1984). 6482839 10.1118/1.595535

[b45] Vaijayanthimala V. . The long-term stability and biocompatibility of fluorescent nanodiamond as an *in vivo* contrast agent. Biomaterials 33, 7794–7802 (2012). 22863379 10.1016/j.biomaterials.2012.06.084

[b46] Massot P. . *In vivo* high-resolution 3D overhauser-enhanced MRI in mice at 0.2 T. Contrast Media Mol. Imaging 7, 45–50 (2012). 22344879 10.1002/cmmi.464

[b47] Saraiva C. . Nanoparticle-mediated brain drug delivery: overcoming blood–brain barrier to treat neurodegenerative diseases. J. Control Rel. 235, 34–47 (2016). 10.1016/j.jconrel.2016.05.044 27208862

[b48] Blanco E., Shen H. & Ferrari M. Principles of nanoparticle design for overcoming biological barriers to drug delivery. Nat. Biotechnol. 33, 941–951 (2015). 26348965 10.1038/nbt.3330 PMC4978509

[b49] Rosen J. E., Chan L., Shieh D.-B. & Gu F. X. Iron oxide nanoparticles for targeted cancer imaging and diagnostics. Nanomedicine 8, 275–290 (2012). 21930108 10.1016/j.nano.2011.08.017

[b50] Zhu B. . Selective magnetic resonance imaging of magnetic nanoparticles by acoustically induced rotary saturation. Magn. Reson. Med. 75, 97–106 (2016). 25537578 10.1002/mrm.25522 PMC4856475

[b51] Haacke E. M., Brown R. W., Thompson M. R. & Venkatesan R. Magnetic Resonance Imaging: Physical Principles and Sequence Design Wiley (1999).

[b52] Moore L. . Biocompatibility assessment of detonation nanodiamond in non-human primates and rats using histological, hematologic, and urine analysis. ACS Nano 10, 7385–7400 (2016). 27439019 10.1021/acsnano.6b00839

[b53] Yuan Y., Chen Y., Liu J. H., Wang H. & Liu Y. Biodistribution and fate of nanodiamonds *in vivo*. Diam. Rel. Mater. 18, 95–100 (2009).

[b54] Zhang X. . Biodistribution and toxicity of nanodiamonds in mice after intratracheal instillation. Toxicol. Lett. 198, 237–243 (2010). 20633617 10.1016/j.toxlet.2010.07.001

[b55] Liersch R., Hirakawa S., Berdel W. E., Mesters R. M. & Detmar M. Induced lymphatic sinus hyperplasia in sentinel lymph nodes by VEGF-C as the earliest premetastatic indicator. Int. J. Oncol. 41, 2073–2078 (2012). 23076721 10.3892/ijo.2012.1665 PMC3583645

[b56] Zbinden G. Fine-Needle Aspiration Biopsy of the Rat Liver: Cytological, Cytochemical and Biochemical Methods Pergamon Press (1980).

[b57] Trencsenyi G. . Metastatic hepatocarcinoma He/De tumor model in rat. J. Cancer 5, 548–558 (2014). 25057306 10.7150/jca.9315 PMC4107231

[b58] Mochalin V. N., Shenderova O., Ho D. & Gogotsi Y. The properties and applications of nanodiamonds. Nat. Nanotechnol. 7, 11–23 (2012). 10.1038/nnano.2011.209 22179567

[b59] Duan X. . Surface-tailored nanodiamonds as excellent metal-free catalysts for organic oxidation. Carbon 103, 404–411 (2016).

[b60] Mi P. . A pH-activatable nanoparticle with signal-amplification capabilities for non-invasive imaging of tumour malignancy. Nat. Nanotechnol. 11, 724–730 (2016). 27183055 10.1038/nnano.2016.72

[b61] Lingwood M. D., Siaw T. A., Chan H. R. & Ross B. D. Hyperpolarized water as an MR imaging contrast agent: feasibility of *in vivo* imaging in a rat model. Radiology 265, 418–425 (2012). 22996746 10.1148/radiol.12111804 PMC3480810

[b62] Abrams D., Trusheim M. E., Englund D. R., Shattuck M. D. & Meriles C. A. Dynamic nuclear spin polarization of liquids and gases in contact with nanostructured diamond. Nano Lett. 14, 2471–2478 (2014). 24754755 10.1021/nl500147b

[b63] Meriles C. A. & Doherty M. W. Generation of spin-polarized currents via cross-relaxation with dynamically pumped paramagnetic impurities. Appl. Phys. Lett. 105, 022403 (2014).

[b64] Bouchard L.-S. . Picomolar sensitivity MRI and photoacoustic imaging of cobalt nanoparticles. Proc. Natl Acad. Sci. USA 106, 4085–4089 (2009). 19251659 10.1073/pnas.0813019106 PMC2657430

[b65] Xing Y. & Dai L. Nanodiamonds for nanomedicine. Nanomedicine UK 4, 207–218 (2009). 10.2217/17435889.4.2.207 19193186

