# Supplementary material for: Nanodiamond-enhanced MRI via in situ hyperpolarization
Source: Nat Commun. 2017 Apr 26;8:15118. doi: 10.1038/ncomms15118 (PMC5414045; doi:10.1038/ncomms15118)
Supplement: Supplementary Information — Supplementary Figures, Supplementary Notes and Supplementary References. [file ncomms15118-s1.pdf]

SUPPLEMENTARY NOTE 1: ADDITIONAL CHARACTERIZATION OF NANODIAMOND SOLUTIONS AND THE OVERHAUSER EFFECT

Nanodiamond (ND) solutions were prepared using high power probe sonication and analyzed by dynamic light scattering (DLS). In Supplementary Fig. 1(a) we present the DLS size distribution obtained for a solution of high-pressure, high-temperature (HPHT) 125 nm ND which shows a median particle size of 125 nm. We note that particle sizes measured with DLS were consistent with manufacturer specifications.

To quantify saturation of the electron paramagnetic resonance (EPR) transition, the nuclear magnetic resonance (NMR) signal enhancement of ND solutions was measured as a function of EPR power. As shown for HPHT 18 nm in Supplementary Fig. 1(b), the enhancement begins to saturate at approximately 30 W. Thus, the Overhauser enhancement is maximized in the spectroscopic measurements presented in Fig. 2, where 49 W of RF power was applied to the EPR resonator.

The relaxivity of ND in water solutions was measured in terms of the longitudinal relaxivity ($1/T_1$) and the transverse relaxivity ($1/T_2$). The relaxivity of various ND types was measured by fitting to the relaxivity equation:

$$1/T_{1,2} = 1/{T^0}_{1,2} + R_{1,2}[\mathrm{ND}] \quad (1)$$

Where $T_{1,2}$ is the ^{1}H relaxation time of the solution with ND, ${T^0}_{1,2}$ is the ^{1}H relaxation time in the absence of ND, $R_{1,2}$ is the relaxivity coefficient, and [ND] is the concentration of ND.

In Fig. 2(d) we present T_1 relaxation data of ND solutions. In Supplementary Fig. 1(c) we present the corresponding T_2 characterization data, obtained in a conventional Hahn echo experiment. T_1 and T_2 values for our stock deionized (DI) water were 2.87 s and 2.60 s respectively.

SUPPLEMENTARY NOTE 2: CALCULATION OF THE COUPLING FACTOR

In Fig. 1(d) we present calculations of the dipolar coupling between ^{1}H nuclei in water and paramagnetic impurities, assumed to be at the ND surface. This calculation is based on a translational Brownian diffusion model, which assumes that the spins are on hard, noninteracting spheres (FFHS - force, free hard sphere model) [1–4].

The dynamics of the ND surface and water molecule can be described by the translational correlation time τ_{t}, defined as:

$$\tau_{\mathrm{t}} = \frac{d^2}{D_{\mathrm{I}} + D_{\mathrm{S}}} \tag{2}$$

where d is the distance of closest approach and D_{I} and D_{S} are the diffusion coefficients for the nuclear spin and electron spin respectively.

Hydration models of ND in aqueous solution show that free diffusion of water occurs at distances greater than 1 nm from the ND surface for particles of sizes up to 200 nm [5]. Within 1 nm of the surface, water diffusion becomes much slower due to the formation of a nanophase of water. Hence, we assume a distance of closest approach between free water molecules and the ND surface of 1 nm and use the diffusion coefficient (D_{I}) of water at room temperature 2.3×10^{-9} m^2 s^{-1} [6]. With dynamic light scattering (DLS) we measured D_{S} of our 125 nm NDs to be 3.4×10^{-12} m^2 s^{-1}, consistent with other reported values [7]. These values give a translational diffusion time of $\tau_{\mathrm{t}} = 430$ ps.

The coupling factor ρ can be expressed in terms of the reduced spectral density function j, [1, 3]:

$$\rho = \frac{5j\left(\omega_{\mathrm{S}}, \tau_{\mathrm{t}}\right)}{7j\left(\omega_{\mathrm{S}}, \tau_{\mathrm{t}}\right) + 7j\left(\omega_{\mathrm{I}}, \tau_{\mathrm{t}}\right)} \tag{3}$$

We use Supplementary Equation 3 to calculate ρ as a function of $\omega_{0\mathrm{S}}\tau_{\mathrm{t}}$. We then plot ρ as a function of magnetic field in Fig. 1(d), by calculating the electron spin resonance frequency $\omega_{0\mathrm{S}}$ for $g_{\mathrm{e}} = -2.002$ and using $\tau_{\mathrm{t}} = 430$ ps.

This calculation assumes that the dipolar interaction is not limited by the relaxation time of the electron, that is $T_{1\mathrm{e}}, T_{2\mathrm{e}} \gg \tau_{\mathrm{t}}$. A typical spin-spin relaxation time for electrons in the surface shell of ND is $T_2 = 300$ ns [8], much greater than τ_{t}.

We have also considered the possibility of the Overhauser effect mediated by rotational diffusion of the nanoparticle. The rotational diffusion coefficient of a spherical particle in solution can be estimated as:

$$\tau_{\mathrm{r}} = \frac{4\pi\eta r^3}{k_{\mathrm{B}}T} \tag{4}$$

Where r is the particle radius, η is the dynamic viscosity of the solution, k_B is the Boltzmann constant and T the temperature. For an 18 nm particle in water this gives $\tau_{\mathrm{r}} = 2.3$ μs for $\eta = 1.002\times10^{-3}$ kg m^{-1} s^{-1} at $T = 293$ K [9]. This timescale, scaling as the cube of radius, suggests that rotational diffusion will play a much smaller role than translation diffusion in

mediating the Overhauser effect in our nanoparticle solutions, however we cannot rule out a contribution, especially for our smaller detonation NDs.

SUPPLEMENTARY NOTE 3: IMPACT OF THE NANOPHASE ON THE OVERHAUSER EFFECT

For HPHT 125 nm ND at 100 mg mL^{-1} we can calculate a leakage factor of $f = 0.87$ from the spin-lattice relaxation measurements in Fig. 2(d) and Eq. 2. However, if we take $\epsilon = -4.0$ from Fig. 2(a), $s = 1$, from Supplementary Fig. 1(b), and $\rho = 0.42$ from Fig. 1(e), then we calculate $f = 0.02$ from Eq. 1. The difference between these two results is indicative of water molecule exchange between the nanophase at the ND surface and free water outside this layer. We expect that ρ, which we calculated for freely diffusing water in Fig. 1(e), is suppressed in the quasistatic nanophase by slow diffusion of water molecules. Meanwhile, ^{1}H nuclei in the nanophase are rapidly relaxed by proximate paramagnetic centers before exchange out of the nanophase, giving the high leakage factor we observed and suppressing the coupling factor observed in freely diffusing water.

Further, we have assumed that the saturation factor in ND solutions takes a maximal value of 1. We note that in systems with multiple components in the EPR spectrum, such as ND, the saturation factor can take values lower than 1 even at high EPR saturation powers [10, 11].

SUPPLEMENTARY NOTE 4: PROBES AND IMAGING

All dynamic nuclear polarization (DNP) experiments were performed with one of three double resonant probes. All probes are based on orthogonal solenoid (NMR) and Alderman-Grant (EPR) resonators. Spectroscopic probe A is optimized for a large electron drive field (B_{1e}), with a high fill factor Alderman Grant resonator at 190 MHz and external solenoid at 276 kHz and was used for all spectroscopic measurements at 6.5 mT. Spectroscopic probe B, used for field sweeps was optimized for high NMR sensitivity over a wide range of frequencies, with an Alderman Grant resonator at 140 MHz. An imaging probe with a 33 mm diameter sample region was built for ND Overhauser-enhanced MRI (OMRI) at $B_0 = 6.5$ mT ($f_{\mathrm{H}} = 276$ kHz, $f_{\mathrm{EPR}} = 190$ MHz). All imaging was performed in our 6.5 mT open-access, human

imaging scanner [12] with the OMRI balanced steady-state free precession (bSSFP) sequence shown in Supplementary Fig. 3(c) [13].

SUPPLEMENTARY FIGURES

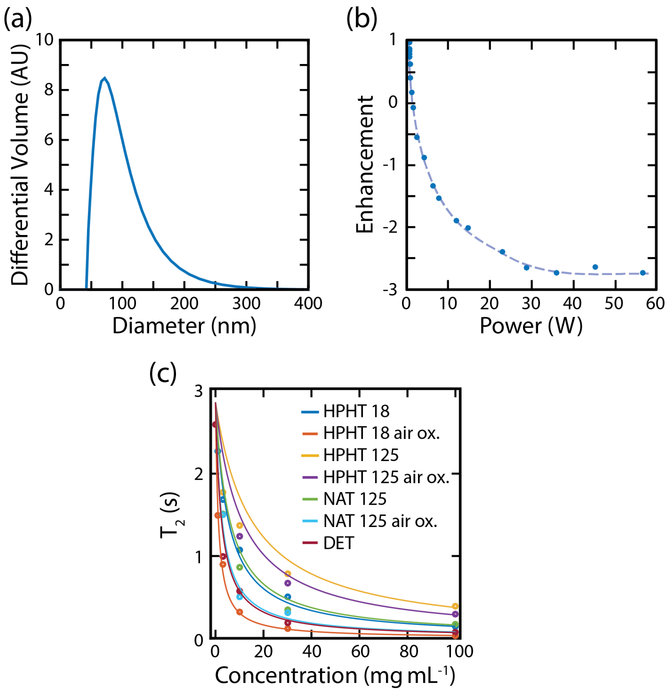

Supplementary Figure 1. **Further characterization of nanodiamond solutions.** (a) Dynamic light scattering characterization of high-pressure, high-temperature (HPHT) 125 nm nanodiamond (ND) sample. The median particle size is 125 nm and the ζ potential in deionized water is -55 mV. (b) ^{1}H enhancement of ND solution as a function of electron paramagnetic resonance (EPR) power. Enhancement is shown for a 100 mg mL^{-1} solution of HPHT 18 nm after a 500 ms EPR pulse at 190 MHz (blue markers). Dotted line is intended as a guide to the eye. (c) T_2 relaxation times of ND solutions. Solid lines are a fit to the concentration dependent relaxivity equation. The fit error on individual T_2 measurements is smaller than the marker size. The T_2 relaxivity coefficients are $6.5 \pm 0.4 \times 10^{-2}$ mL s^{-1} mg^{-1} for HPHT 18 nm (blue), $2.9 \pm 0.1 \times 10^{-1}$ mL s^{-1} mg^{-1} for air oxidized HPHT 18 nm (orange), $2.3 \pm 0.4 \times 10^{-2}$ mL s^{-1} mg^{-1} for HPHT 125 nm (yellow), $3.2 \pm 0.5 \times 10^{-2}$ mL s^{-1} mg^{-1} for air oxidized HPHT 125 nm (purple), $5.8 \pm 1.1 \times 10^{-2}$ mL s^{-1} mg^{-1} for natural (NAT) 125 nm (green), $1.3 \pm 0.2 \times 10^{-1}$ mL s^{-1} mg^{-1} for air oxidized NAT 125 nm (light blue) and $1.4 \pm 0.1 \times 10^{-1}$ mL s^{-1} mg^{-1} for detonation (DET, red).

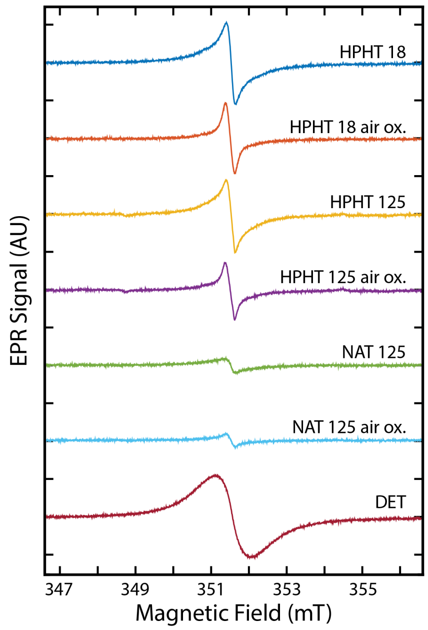

Supplementary Figure 2. **X-band EPR Spectra of Nanodiamond Solutions.** Electron paramagnetic resonance (EPR) spectra of nanodiamonds (NDs) in 100 mg mL^{-1} solutions of deionized water. High-pressure, high-temperature (HPHT) 18 nm (blue), air oxidized HPHT 18 nm (orange), HPHT 125 nm (yellow), air oxidized HPHT 125 nm (purple), natural (NAT) 125 nm (green), air oxidized NAT 125 nm (light blue) and detonation (DET, red). Spectra are offset for clarity. All spectra are well fit by a model employing broad and narrow spin-1/2 components, with the exception of the HPHT 125 nm sample, which shows an additional component due to P1 centres. These P1 centres are known to be due to substitutional nitrogen atoms in the crystalline core of the ND [14] and we expect that their interaction with water molecules will be much smaller than that of surface defects. We note that detonation ND only shows the broad component from our two spin fit and that natural ND shows a much smaller defect concentration compared to other ND types.

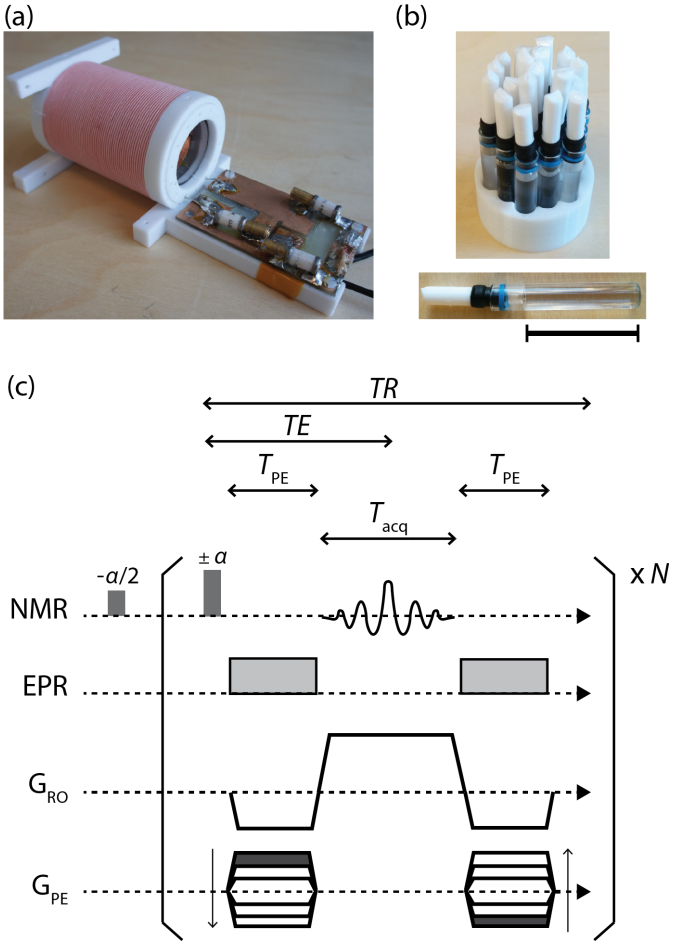

Supplementary Figure 3. **Details of OMRI setup.** (a) Double resonant Overhauser-enhanced MRI (OMRI) imaging probe. The external solenoid was used for nuclear magnetic resonance (NMR) acquisition at 276 kHz. An internal Alderman-Grant resonator was used to saturate the electron paramagnetic resonance (EPR) resonance at 190 MHz. (b) Phantom with vials of ND solution and vials of water, used for imaging in Fig. 3. Scale bar is 20 mm in length. (c) Balanced steady-state free precession (bSSFP) OMRI imaging sequence used for imaging. Relative timing of NMR pulses, EPR pulses, readout gradient (G_{RO}) and phase encode gradient (G_{PE}) are shown. Imaging parameters were: repetition time (TR) = 86 ms, echo time (TE) = 43 ms, acquisition time (T_{acq}) = 28 ms, phase encode time (T_{PE}) = 22.5 ms and α = 70°.

SUPPLEMENTARY REFERENCES

[1] Armstrong, B. D. & Han, S. Overhauser dynamic nuclear polarization to study local water dynamics. *J. Am. Chem. Soc.* **131**, 4641–4647 (2009).

[2] Tóth, É., Helm, L. & Merbach, A. E. Relaxivity of MRI Contrast Agents. *Top. Curr. Chem.* **221**, 61–101 (2002).

[3] Hwang, L.-P. & Freed, J. H. Dynamic effects of pair correlation functions on spin relaxation by translational diffusion in liquids. *J. Chem. Phys.* **63**, 4017–4025 (1975).

[4] Freed, J. Dynamic effects of pair correlation functions on spin relaxation by translational diffusion in liquids. II. Finite jumps and independent T_1 processes. *J. Chem. Phys.* **68**, 4034–4037 (1978).

[5] Korobov, M., V., A. N., Bogachev, A. G., Rozhkova, N. N. & Osawa, E. Nanophase of water in nano-diamond gel. *J. Phys. Chem. C* **111**, 7330–7334 (2007).

[6] Holz, M., Heil, S. R. & Sacco, A. Temperature-dependent self-diffusion coefficients of water and six selected molecular liquids for calibration in accurate ^{1}H NMR PFG measurements. *Phys. Chem. Chem. Phys.* **2**, 4740–4742 (2000).

[7] Neugart, F. *et al.* Dynamics of diamond nanoparticles in solution and cells. *Nano Lett.* **7**, 3588–3591 (2007).

[8] Yavkin, B. V. *et al.* Defects in Nanodiamonds: Application of High-Frequency cw and Pulse EPR, ODMR. *Applied Magnetic Resonance* **45**, 1035–1049 (2014).

[9] Kestin, J., Sokolov, M. & Wakeham, W. A. Viscosity of Liquid Water in the Range of -8°C to 150°C. *J. Phys. Chem. Ref. Data* **7**, 941–948 (1978).

[10] Armstrong, B. D. *et al.* Portable X-band system for solution state dynamic nuclear polarization. *J. Magn. Reson.* **191**, 273–281 (2008).

[11] Ravera, E., Luchinat, C. & Parigi, G. Basic facts and perspectives of Overhauser DNP NMR. *J. Magn. Reson.* **264**, 78–87 (2016).

[12] Sarracanie, M. *et al.* Low-Cost High-Performance MRI. *Sci. Rep.* **5**, 15177 (2015).

[13] Sarracanie, M., Armstrong, B. D., Stockmann, J. & Rosen, M. S. High speed 3D overhauser-enhanced MRI using combined b-SSFP and compressed sensing. *Magn. Reson. Med.* **71**,

735–745 (2013).

[14] Yavkin, B. V., Mamin, G. V., Gafurov, M. R. & Orlinskii, S. B. Size-dependent concentration of N^0 paramagnetic centres in HPHT nanodiamonds. *Magn. Reson. Solids* **17**, 15101 (2015).
